# Hearing in Complex Environments: Auditory Gain Control, Attention, and Hearing Loss

**DOI:** 10.3389/fnins.2022.799787

**Published:** 2022-02-10

**Authors:** Benjamin D. Auerbach, Howard J. Gritton

**Affiliations:** ^1^Department of Molecular and Integrative Physiology, Beckman Institute for Advanced Science and Technology, University of Illinois at Urbana-Champaign, Urbana, IL, United States; ^2^Neuroscience Program, University of Illinois at Urbana-Champaign, Urbana, IL, United States; ^3^Department of Comparative Biosciences, University of Illinois at Urbana-Champaign, Urbana, IL, United States; ^4^Department of Bioengineering, University of Illinois at Urbana-Champaign, Urbana, IL, United States

**Keywords:** adaptation, gain control, attention, auditory scene analysis, cocktail party problem, hearing loss

## Abstract

Listening in noisy or complex sound environments is difficult for individuals with normal hearing and can be a debilitating impairment for those with hearing loss. Extracting meaningful information from a complex acoustic environment requires the ability to accurately encode specific sound features under highly variable listening conditions and segregate distinct sound streams from multiple overlapping sources. The auditory system employs a variety of mechanisms to achieve this auditory scene analysis. First, neurons across levels of the auditory system exhibit compensatory adaptations to their gain and dynamic range in response to prevailing sound stimulus statistics in the environment. These adaptations allow for robust representations of sound features that are to a large degree invariant to the level of background noise. Second, listeners can selectively attend to a desired sound target in an environment with multiple sound sources. This selective auditory attention is another form of sensory gain control, enhancing the representation of an attended sound source while suppressing responses to unattended sounds. This review will examine both “bottom-up” gain alterations in response to changes in environmental sound statistics as well as “top-down” mechanisms that allow for selective extraction of specific sound features in a complex auditory scene. Finally, we will discuss how hearing loss interacts with these gain control mechanisms, and the adaptive and/or maladaptive perceptual consequences of this plasticity.

## Introduction

Auditory scene analysis— the ability to segregate specific sound features from multiple overlapping sources— is essential for extracting meaningful information from a complex sound environment (Bregman, 1990). The classic example of this problem is the cocktail party effect, where a listener can selectively focus on one specific speaker while filtering out a range of other stimuli (Cherry, 1953; Bregman, 2008). While the cocktail party problem represents a particularly challenging situation for the auditory system, as both the target and background sounds are comprised of similar acoustic features, most behaviorally-relevant sounds (such as a person talking) occur against a background of everyday noise (e.g., traffic noise, a loud TV, etc.). Thus, adapting to noisy environments is a fundamental feature of the auditory system important for a range of listening conditions (Willmore et al., 2014; King and Walker, 2020). Understanding how the auditory system adapts to complex sound environments has important clinical implication as well, as individuals with age-related hearing loss or other hearing impairments often have great difficulties listening in noise, even when cochlear amplification is accounted for (Johannesen et al., 2016). How the auditory system solves the problem of auditory scene analysis remains incompletely understood.

Like humans, many animals— such as birds (Hulse et al., 1997), frogs (Endepols et al., 2003), and other mammals (Ma et al., 2010; Chapuis and Chadderton, 2018; Noda and Takahashi, 2019)— are capable of listening to a single sound source in a mixture of sources. Here we will discuss recent evidence from animal and human literature regarding the neurophysiological mechanisms for auditory scene analysis and hearing in complex environments. In particular, we will focus on gain control mechanisms— adjustments to the slope and dynamic range of neural input–output (I/O) relationships— that allow neurons to actively regulate their response sensitivity to the current environmental or behavioral demands (Robinson and McAlpine, 2009; Ferguson and Cardin, 2020). First, we will discuss how the auditory system adapts its response properties to changes in the overall distribution of incoming stimulus features. This bottom-up adaptation to stimulus statistics allows for extraction and invariant representation of key auditory features used to segregate sound sources in complex and continually changing acoustic environments. Next, we will discuss top-down contextual and attentional gain control mechanisms that can highlight behaviorally relevant sound information while selectively filtering distracting sources, even with overlapping acoustic features. Finally, we will examine how the central auditory system adapts to cochlear hearing loss and how this compensatory plasticity can have both adaptive and maladaptive consequences for sound perception and listening in complex auditory environments.

## Bottom-Up Adaptation to Sound Statistics

**FIGURE 1:**
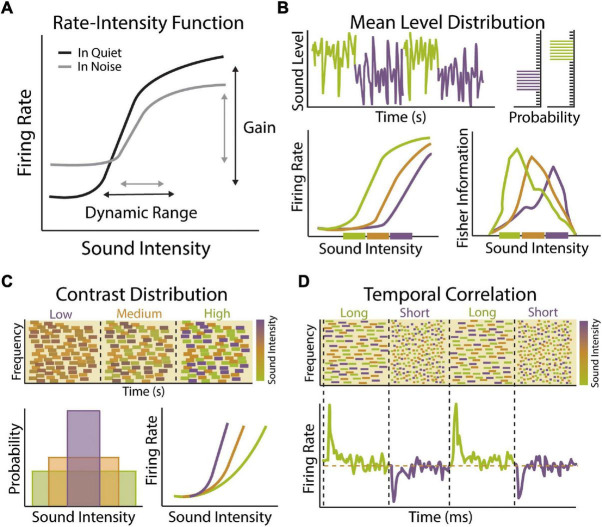
Stimulus statistic adaptation in the auditory system. **(A)** Example rate-intensity function showing input–output relationship of sound intensity and auditory neuron firing rate in quiet (black) or in the presence of background noise (gray). Auditory neurons encode sound level information through changes in mean firing rate. The neuron’s response gain is defined as the rate at which neuronal firing increases as a function of increasing sound level input. The dynamic range of a neuron is defined as the range of stimulus values encoded by a neuron though changes in its firing rate. A majority of auditory neurons exhibit low thresholds with firing rates that saturate to low or moderate sound levels, limiting their dynamic range. Both the gain and dynamic range of auditory neurons become compressed in the presence of background noise, which can be compensated for in part by adaptation to sound level statistics. **(B)** Example of dynamic range adaptation in a central auditory neuron or auditory nerve (AN) fiber. Top: Switching stimulus with high probability density region (HPR) at low (green) or high (purple) sound intensity levels. Within each environment, the range of intensities is drawn from a statistically defined high probability region confined to a narrow range of intensity while the remaining stimuli are drawn from a broader range of intensities outside the HPR (top right). Bottom: Auditory neurons adapt their threshold and dynamic range so that they are most sensitive to sound intensities within in the HPR, indicated by thick colored bars on *x*-axis (bottom left). Fisher information measure of coding accuracy for rate-intensity functions indicates that dynamic range adaptation acts to improve accuracy of sound intensity coding for sound levels most likely to be encountered in current environment (bottom right). Schematized data adapted from Dean et al. (2005). **(C)** Example of contrast gain control in a central auditory neuron. Top: Spectrogram of dynamic random chord (DRC) stimuli with low, medium or high contrast in sound intensity levels. *Bottom:* Theses spectrotemporally complex stimuli have same mean sound level but different sound level variance (bottom left). Neuronal gain shifts with changes in contrast (bottom right). Response gain is steep in low contrast environments (purple), allowing neurons to be sensitive to small changes in sound intensity, but becomes progressively shallower in medium (orange) and low (green) contrast environments, allowing neurons to maintain sensitivity to a larger range of intensities. Schematized data adapted from Rabinowitz et al. (2011). **(D)** Example of gain adaptation to changes in temporal correlation of sounds in central auditory neuron. Top: A series of DRCs with dynamically changing temporal correlation structure. Bottom: Auditory neurons respond to transition in temporal correlation structure by brief increases or decreases in firing rate, followed by steady state firing rates (orange dashed line). These transient changes in firing rate are indicative of adaptation to statistical change in stimulus, indicating that auditory neurons can adapt to the temporal dynamic range of the inputs to preserve encoding efficiency under varying statistical constraints without changing overall activity levels. Schematized data adapted from Natan et al. (2016).

Most natural sounds, including human speech, are characterized by dynamic changes in acoustic energy across spectral and temporal domains (Davenport, 1952; Singh and Theunissen, 2003; Santoro et al., 2014). In order to efficiently analyze an auditory scene and accurately represent the vast range of sounds encountered in the world, auditory neurons must be able to continually adapt their response properties to the prevailing acoustic environment. There is ample evidence that neural representations of sound are sensitive to statistical regularities in the acoustic environment (Winkler et al., 2009). For instance, many neurons across the auditory neuraxis exhibit stimulus-specific adaptation (SSA), in that they become less responsive to frequently occurring or repetitive stimuli but retain their sensitivity to rare stimuli, allowing for an intrinsic capacity to selectively encode unpredictable or novel sounds (Ulanovsky et al., 2003; Nelken, 2014). In addition to adapting to their own stimulus history, auditory neurons can also modify their response properties to match the statistics of the entire distribution of sounds encountered in the environment. Auditory neurons adapt their dynamic range and gain in response to a variety of stimulus statistics (Figure 1), including: mean sound level (Dean et al., 2005; Wen et al., 2009; Barbour, 2011), sound level variance or contrast (Nagel and Doupe, 2006; Rabinowitz et al., 2011; Willmore et al., 2014), interaural sound cues (Dahmen et al., 2010; Stange et al., 2013), and spectral-temporal correlations (Kvale and Schreiner, 2004; Natan et al., 2016; Homma et al., 2020). In this manner, neuronal responses are continuously rescaled to match dynamically changing sound conditions while maintaining overall firing rates across stimuli with different statistics. This adaptation to sound statistics enables auditory neurons to efficiently encode a wide-range of stimulus features under highly variable conditions and may be an effective mechanism for generating relatively invariant sound representations that are robust to the presence of background noise. Below we will discuss evidence for different forms of stimulus statistic adaptation as well as our current understanding of the neurophysiological mechanism and perceptual consequences of these adaptations.

### Dynamic Range Adaptation

Natural acoustic scenes are characterized by stimuli that can vary over a wide range of sound levels, roughly 10–12 orders of magnitude (Baccus, 2006; Robinson and McAlpine, 2009). The auditory system maintains a remarkable sensitivity to small differences in sound level over this enormous range of intensities despite the relatively restricted dynamic range of individual auditory neurons, typically 30–50 dB SPL (Figure 1A) (Sachs and Abbas, 1974; Viemeister, 1988). This so-called dynamic range problem is compounded by noisy environments that act to increase the steady-state firing rate of auditory neurons, thereby limiting their dynamic range even further (Figure 1A) (Costalupes et al., 1984; Young and Barta, 1986). One potential solution to the dynamic range problem is to have distinct subsets of auditory neurons with different thresholds and dynamic ranges, such that combining or stitching together individual response functions would allow for representation of intensities across the full range of hearing at the population level (Barbour, 2011). Dynamic range stitching is observed to some degree at the level of the auditory nerve (AN), where fibers can be classified into at least three distinct subsets based on their response threshold and spontaneous firing rates (SR) (Evans, 1972; Sachs and Abbas, 1974; Liberman, 1978). Low-SR fibers have high thresholds and large dynamic ranges, medium-SR fiber have intermediate thresholds and dynamic range, while high-SR fibers have low thresholds and narrow dynamic range. Thus, while individual AN fibers have a restricted dynamic range, their sensitivity is distributed across a range of intensities. Moreover, the high threshold and larger dynamic range of low-SR fibers make them better suited for encoding intensity at higher sound levels and more resistant to background noise, suggesting they may be important for hearing in a noisy environment (Costalupes et al., 1984). Dynamic range stitching is also observed in the central auditory system, with a subset of central auditory neurons exhibiting non-monotonic response functions that respond best to a particular sound level rather than exhibiting a constant increase in firing rate with increasing sound intensity (Suga and Manabe, 1982; Phillips et al., 1994; Sadagopan and Wang, 2008). However, the vast majority of auditory neurons have thresholds and dynamic ranges that are still heavily skewed toward lower sound intensities and it is therefore unlikely that dynamic range stitching can fully account for the maintenance of consistent sound level sensitivity across the entire range of hearing (Sachs and Abbas, 1974; Watkins and Barbour, 2011). It is becoming increasingly clear that a major mechanism for solving the dynamic range problem is that individual auditory neurons can dynamically adapt their threshold and dynamic range to compensate for changes in mean stimulus level, thereby maintaining maximum sensitivity over the most commonly encountered sound levels in the prevailing acoustic environment (Figure 1B) (Dean et al., 2005).

Dynamic range adaptation to mean sound level is observed across multiple levels of the auditory system, most notably at the AN (Wen et al., 2009), inferior colliculus (IC) (Dean et al., 2005, 2008), and auditory cortex (ACx) (Watkins and Barbour, 2008). When sounds are drawn from a distribution with a high probability of loud sounds, such that the mean sound level is high, neurons shift their dynamic range upward, increasing their sensitivity to louder sounds (Figure 1B). Sound-level adaptation is thus compensatory, so that neuronal responses are relatively invariant to changes in background level. The degree of dynamic range adaptation observed in the IC is higher than at the level of the AN, suggesting that adaptation in the auditory midbrain is only partially accounted for by changes occurring in the periphery. This suggest that additional adaptation is occurring within the IC and potentially in the auditory brainstem as well. Indeed, neurons in the ventral cochlear nucleus (VCN) of the brainstem have been shown to adapt their threshold and dynamic range to the presence of background noise (May and Sachs, 1992). However, these studies employed a stationary background noise and experiments using the same dynamic switching stimulus used to measure mean level adaptation in the AN and IC have not been performed at the level of the brainstem. Similar magnitudes of adaptation are seen in subcortical and cortical neurons, suggesting that many features of mean level adaptation in cortex are inherited from lower levels of the auditory system. However, there are unique features of adaptation in the ACx, such as a subset of cortical neurons with non-monotonic rate-level functions that do not undergo adaptive recoding and are thus maladapted to high level sounds but preserve coding accuracy for quiet sounds even in the context of high sound level environments (Watkins and Barbour, 2008).

### Contrast Gain Control

The auditory system is not only exposed to a wide range of sound levels, but any acoustic scene may be comprised of a relatively large or restricted subset of intensities across this spectrum. Thus, in addition to adapting to mean sound level, auditory neurons must be able to modulate their response properties to changes in the variance or contrast of sound levels present in the environment (Figure 1C). Contrast-invariant tuning is one of the most well-characterized examples of gain modulation observed across sensory systems (Finn et al., 2007; Olsen and Wilson, 2008; Rabinowitz et al., 2011) and contrast gain control in the auditory system is thought to be an important physiological mechanisms for encoding sound stimuli in noisy background conditions (Willmore et al., 2014). When an auditory neuron is exposed to a wide range of sound intensities, such that the contrast of the input is high, the gain of that neuron is low (Figure 1C). In this manner, the neuron has a broader dynamic range that is relatively insensitive to changes in sound level. When the contrast of the input is low, the gain of the neuron increases, making it more sensitive to small changes in intensity. Thus, like mean level adaptations, contrast gain control is compensatory, allowing neurons to adjust their gain in a manner that allows for representations that are relatively invariant to the level of background noise. Such adaptation allows for sounds that are structurally similar but with different contrast levels to be represented in a similar manner. It should also be noted that auditory neurons adjust their response properties to higher-order stimulus statistics like skewness or kurtosis as well (Kvale and Schreiner, 2004). Studies in ferrets have found that contrast gain control is more complete in the ACx compared to subcortical stations (Rabinowitz et al., 2013). However, more recent work in mice has found similar levels of contrast gain adaptation in the ACx, auditory thalamus (medial geniculate body; MGB), and IC (Lohse et al., 2020). Notably, the authors did find that adaptation time constants become longer at ascending levels of the auditory system, resulting in progressively more stable representations. Thus, there may be progressive changes to contrast gain control along the ascending auditory pathway, similar to that observed with mean level adaptation.

The combined effect of dynamic range adaptation and contrast gain control is to minimize the influence of background noise on auditory feature encoding. Indeed, by the level of the ACx, adaptation to mean level and contrast enables speech sounds to be represented in a way that is robust to the presence of background noise (Rabinowitz et al., 2013; Mesgarani et al., 2014b). However, it is important to note that adaptation to other acoustic features beyond sound intensity is likely important for speech perception and auditory scene analysis as well. Spectral features are a fundamental component of communication signals in mammalian vocalizations (Suga et al., 1983; Kadia and Wang, 2003). Human speech is comprised of several harmonic features and the use of these features can be helpful for identifying a speaker in a complex environment (Ehret and Riecke, 2002). Speech also varies in its temporal profile, including elements of fast temporal modulation and slower changes associated with periodicity of the speech signal, and the temporal structure of human vocalizations plays a crucial role in speech comprehension (Rosen, 1992; Shannon et al., 1995; Hickok and Poeppel, 2007). Frequency-specific adaptations have been observed in the human ACx that depend on the spectral range of acoustic stimuli, suggesting that there are neural adjustments to spectral stimulus statistics of sound stimuli (Herrmann et al., 2014). ACx neurons also display gain adaptations to changes in the temporal properties of sound input, allowing them to maintain their dynamic range across a range of temporal correlations (Natan et al., 2016) and use non-linear sensitivity to temporal and spectral content for adaptation (Figure 1D) (Angeloni and Geffen, 2018). Thus, in addition to sound level adaptations, neuronal adjustments to spectral and temporal sounds properties are also likely important for neural representation of speech in different background conditions (Ding and Simon, 2013; Mesgarani et al., 2014b; Khalighinejad et al., 2019).

### Adaptation in Sound Localization

In a natural environment, multiple sound sources often originate from different locations and being able to identify the spatial location of distinct sound sources is a key component to auditory scene analysis. Binaural cues, such as interaural time (ITD) and level (ILD) differences, are essential for localizing sounds in space. The medial superior olive (MSO) and lateral superior olive (LSO) of the auditory brainstem are the initial sites of ITD and ILD processing in the mammalian auditory system, respectively. These brainstem nuclei contain coincidence detecting neurons that encode ITD and ILD differences by comparing the timing of converging inputs from the ipsilateral and contralateral ear with submillisecond precision (Park et al., 2004; Grothe et al., 2010). Because of the degree of precision required for these computations, and the fact that accurate representation of absolute stimulus values may be more important for sound-source localization than for other acoustic features like sound level, traditional models of sound localization have proposed that ITDs and ILDs are encoded via a fixed labeled-line mechanism resulting in a hard-wired place code or map of auditory space (Jeffress, 1948; Grothe and Koch, 2011). However, it has now been shown that sound localization cues and spatial perception are also subject to short-term adaption based on prior stimulus history (Phillips and Hall, 2005; Vigneault-MacLean et al., 2007; Dahmen et al., 2010; Stange et al., 2013). Indeed, coincidence-detector neurons in both the MSO and LSO exhibit dynamic gain adaptations driven by feedback loops in early parts of the binaural pathway that modulate their sensitivity to ITD and ILD differences in response to prior activity levels (Finlayson and Adam, 1997; Magnusson et al., 2008; Park et al., 2008; Stange et al., 2013; Lingner et al., 2018). Adaption to sound source localization is observed in downstream auditory structures as well. By presenting sound sequences in which ILDs rapidly fluctuate according to a Gaussian distribution, it was shown that IC neurons also adapt the dynamic range and gain of their ILD-rate functions to match the mean and variance of the stimulus distribution (Dahmen et al., 2010). Thus, adaptive gain control mechanisms can also modulate the population code for auditory space in a stimulus history dependent manner.

### Perceptual Consequences of Stimulus Statistic Adaptation

The above studies demonstrate that the auditory system uses multiple adaptive coding strategies to most efficiently represent and extract features from the sound environment. However, elucidating the perceptual consequences of these adaptations is crucial for determining if and how they facilitate our ability to analyze an auditory scene. Several recent studies have found that perceptual adaptations to stimulus statistics in humans parallel neurophysiological adaptations in animal models using near identical paradigms (Dahmen et al., 2010; Stange et al., 2013; Lohse et al., 2020). For instance, there is a close correspondence between changes to the perceived laterality of a stimulus in humans and adaptations to ILD-rate functions in the IC of ferrets when both are presented with noise sequences with rapidly fluctuating ILDs (Dahmen et al., 2010). Likewise, acuity in an intensity discrimination task is rapidly adjusted with changes to sound contrast in humans and the strength of this perceptual contrast adaptation could be predicted from physiological contrast adaptation observed in mice (Lohse et al., 2020). Chronic *in vivo* recordings from the ACx of mice trained to detect a target sound in background noise shortly after a change in the background contrast have provided some of the first evidence that cortical gain modulation and sound detection behavior are modulated by contrast in a parallel manner in the same subjects (Angeloni et al., 2021). This study found that ACx activity is necessary for detection of targets in background noise and that inter-subject variability in the magnitude of contrast gain control observed in the ACx predicted behavioral performance. These findings provide evidence that adaptive coding in the ACx has direct implications on perceptual behavior. In contrast, single unit recordings from the IC of macaques performing a masked tone detection task found that, despite observing dynamic range adaptation in the IC of these animals, behavioral detection thresholds were not affected by this neuronal adaptation (Rocchi and Ramachandran, 2018). Likewise, MEG and EEG studies have found evidence for dynamic range adaptation in the ACx of humans but parallel behavioral studies found that perceptual sensitivity to sound level was actually affected in an opposite manner than predicted by dynamic range adaptation, with increased sensitivity to sound intensities in the low probability region of the intensity distribution rather than high (Simpson et al., 2014; Herrmann et al., 2020). Thus, while there is growing evidence that adaption to stimulus statistics does influence perception, more work is needed to determine how different forms of adaptation across levels of the auditory system contribute to sound perception and auditory scene analysis.

Many studies have now shown that auditory neurons adapt their response properties to a range of stimulus statistics and tremendous progress has been made in the neurophysiological characterization of these bottom-up adaptations. However, there are many open questions that remain to be addressed. For instance, while the above studies indicate that adaptive coding is gradually built along the auditory pathway, the relative contributions of different auditory structures remain incompletely understood. More studies utilizing simultaneous recordings from multiple auditory regions are needed to determine how different forms of adaptation emerge along the ascending auditory pathway. Indeed, a recent study using this approach has uncovered a previously underappreciated role for subcortical processing in contrast gain control (Lohse et al., 2020). Second, the underlying cellular and circuit mechanisms driving adaption to sound statistics need to be fully elucidated, as will be discussed in subsequent sections. This knowledge is essential for understanding the biophysical constraints on theses adaptive processes as well as for generating novel strategies for manipulating these processes to better investigate their contribution to auditory scene analysis. Finally, more studies performing neurophysiological recordings from actively behaving animals are necessary to directly assess the impact of bottom-up adaptions on perception (Angeloni et al., 2021). One difficulty in assessing the perceptual consequences of bottom-up adaptations is that, in most cases, measuring behavioral sensitivity to changes in stimulus statistics requires subjects to be engaged in a perceptual decision-making task. Task engagement itself will invoke a multitude of adaptive changes in the auditory system, as will be discussed in the next section.

## Top-Down Contributions to Auditory Scene Analysis

Bottom-up adaptations to the prevailing sound statistics enable the auditory system to more efficiently encode target sounds in complex or noisy environments, particularly when the statistics of foreground and background sounds are distinct (Figure 1). However, background sounds that share acoustic features or statistical properties that significantly overlap with the signals of interest, such as is the case for the cocktail party problem, pose unique challenges for auditory scene analysis and additional mechanisms must exist to selectively extract specific sound sources from structurally similar background noise (King and Walker, 2020). Attention is a cognitive process by which organisms filter the most relevant behavioral information from their environment to enhance perception of one particular stimulus over another. Selective attention has been proposed to contribute to auditory scene analysis by acting as a form of sensory gain control, enhancing the representation of an attended sound source while suppressing responses to unattended sounds (Fritz et al., 2007; Kerlin et al., 2010; Zion Golumbic et al., 2013). This process can occur when the stimulus itself directs attention through enhanced salience, referred to as bottom-up or “pop-out” attention (Kayser et al., 2005), or can be endogenously generated through top-down or “task-modulated” processes. The focus of this section will be to discuss these top-down mechanisms and how selective attention contributes to gain modulation, feature selection, and stream separation in the auditory system, which work in concert to improve auditory scene analysis. First, we will discuss the growing body of evidence from animal studies showing that sensory encoding is fundamentally modulated by behavioral state. Then, we will discuss evidence that task-engagement, a proxy for attention in animal models, is associated with receptive field changes that act to maximize encoding of task-relevant information. Finally, we will discuss evidence from human studies showing that selective attention does indeed influence perception and listening performance in complex auditory environments.

### Behavioral State and Attentional Modulation of Sensory Processing

Behavioral states have strong influences on neuronal responses associated with sensory processing (Bennett et al., 2014; Lee et al., 2014). Early studies that monitored pupil dilation as a proxy for arousal found that cortical neurons in sensory regions are strongly modulated by dilation onset and that neuronal firing rates correlate with the level of dilation even in the absence of sensory input (Iriki et al., 1996). These results suggest that internal state changes may influence how we process incoming sensory information. Indeed, performance on a tone-in-noise detection task has been shown to be highly state-dependent, with peak behavioral performance being associated with intermediate levels of arousal (McGinley et al., 2015a). These intermediate arousal states coincided with periods of stable hyperpolarization in auditory cortical neurons. Subsequent studies using optogenetic manipulations of inhibitory interneuron populations found that such hyperpolarized states increase encoding specificity by augmenting the threshold for responsivity of excitatory neurons while simultaneously narrowing the frequency tuning properties in principle cell populations (Hamilton et al., 2013; Aizenberg et al., 2015; Phillips and Hasenstaub, 2016). Selective attention also profoundly impacts behavioral sensitivity to sensory stimuli and is in fact operationally defined as an improvement in psychophysical performance for attended versus unattended stimuli (e.g., Carrasco, 2011). Neurophysiological studies have revealed that attentional effects on sensory processing are due at least in part to gain modulation that increases stimulus-evoked response size (Mitchell et al., 2007; Maunsell, 2015). Indeed, tone-evoked responses in the ACx are greater in animals performing a tone-detection task compared to passive listening conditions, indicative of attentional gain modulation (Francis et al., 2018). Taken together, these findings offer insight into how behavioral state changes influence sensory processing irrespective of the sound statistics being conveyed to the sensory system.

While behavioral state and attentional gain increases enhance the magnitude of sensory-evoked responses, it is important to note that a non-selective increase in neuronal activity is not necessarily beneficial to stimulus detection. Rather, attention appears to enhance feature encoding by modulating not only the magnitude of the sensory stimulus but also the spontaneous activity or “noise” of neural responses (Harris and Thiele, 2011). Background noise can include non-stimulus specific activity represented by highly correlated neurons that act to reduce the amount of information that can be encoded for a particular stimulus or through competing distractors in the stimulus field (Zohary et al., 1994; Fries et al., 2001; Moreno-Bote et al., 2014). Selective attention not only increases stimulus-evoked responses, but also reduces the effect of intrinsic background noise, thereby enhancing signal-to-noise ratios for sensory representations and decreasing trial-to-trial variability (Mitchell et al., 2009; Downer et al., 2017; Francis et al., 2018). Selective attention simultaneously reduces variability and noise correlations across populations of cortical neurons in large part by reducing low frequency firing rate correlations to produce a sparse and temporally reliable code (Mitchell et al., 2009; Francis et al., 2018). This appears to be the case for arousal-dependent changes in sound processing as well, as changes in pupil diameter produce bi-modal effects on spontaneous and sensory-evoked activity that improve signal-to-noise ratios of sound-evoked responses (McGinley et al., 2015b). Such findings argue that reduction in spontaneous neural activity is as critical to feature discrimination as gain modulated increases in firing rate. Indeed, attentional control associated with the act of behavioral engagement appears to enhance feature encoding by altering the spontaneous activity of cortical circuits prior to sensory processing. For example, the process of self-directed trial initiation decreases the rate of spontaneous activity in the ACx of rats performing a tone-detection task and optogenetic disruption of cortical activity before tone presentation acts to impair performance (Carcea et al., 2017). Thus, attention has the effect of both increasing responsiveness to a target stimulus while simultaneously reducing the influence of background activity and distracting inputs, which together act to stabilize sensory representations and promote feature tracking in complex environments.

### Task-Dependent Modulation of Sound Feature Encoding

**FIGURE 2:**
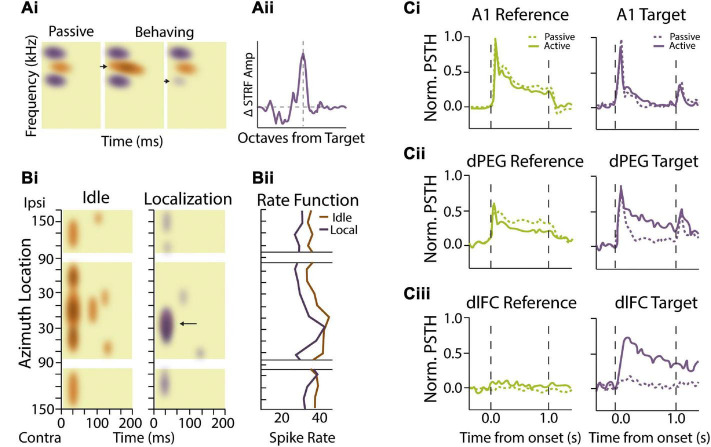
Attentional and task-dependent modulation of sound feature encoding. **(Ai,ii)** Spectrotemporal receptive fields (STRFs) in auditory cortical neurons change based on task engagement and target frequency. **(Ai)** Example STRF showing enhanced sensitivity (orange) and sideband inhibition (purple) during passive presentation of broadband temporally orthogonal ripple combinations (TORC) stimuli (left). Performance of a tone detection task with peak STRFs near the target frequency (arrow) enhances the excitatory region in the STRF during behavior (middle). When tonal targets were presented with frequencies that coincided with inhibitory STRF, (arrow) the STRF showed local decreases or elimination of inhibitory sidebands (right). **(Aii)** Summarized data showing that STRF plasticity adaptation effects are most substantial when near the target frequency with facilitation occurring over ∼1 octave from the target stimulus. Schematized data adapted from Fritz et al. (2003). **(Bi,ii)** Spatial sensitivity modulated by task performance. **(Bi)** Heat maps demonstrating primary auditory cortex (A1) neural activity as a function of time (horizontal axis) and stimulus location (vertical axis) from a single behavioral session. This neuron shows burst activity at sound onset and is strongly responsive to probe trials originating from all locations during idle conditions (non-task performing condition). During the sound localization task where the cat is rewarded for discriminating changes in elevation, neural responses become more selective for probe trial origin, responding best to stimuli located between contralateral 10° and 50°. Arrow indicates increased specificity for this unit at the spatial localization. Colors indicate changes in mean intensity firing rates for the two conditions. **(Bii)** Rate functions in response to sound onset are shown to the right for the passive and sound location task conditions as a function of stimulus location. Schematized data adapted from Lee and Middlebrooks (2011). **(Ci,ii,iii)** Effects of task performance on auditory responsivity in auditory and frontal cortices. **(Ci)** Average behavior-dependent change in reference (green) and target (purple) responses in A1. Reference targets included TORC or narrowband white noise stimuli while targets consisted of pure tones. Dashed lines represent pre-task passive responses while solid lines represent task-engaged response. The average reference and target response as measured by normalized peri-stimulus time histograms (PSTH) amplitude were not significantly different between passive and behavior conditions. **(Cii)** Target and reference comparison for dorsal posterior ectosylvian gyrus (dPEG) of the ferret which is a belt region receiving A1 input. dPEG shows an average target response augmentation during task performing conditions. **(Ciii)** Target and reference PSTH comparison for dorsal lateral frontal cortex (dlFC), an executive region important for cue-directed behavior. dlFC neurons show almost no responsivity during passive conditions for either target or reference stimuli; however, they are strongly regulated by the target exclusively during behavior. Schematized data adapted from Atiani et al. (2014).

The above studies suggest that auditory responses rely not only on the external sounds reaching the ear, but also on the behavioral context and internal state of the subject. While it is clear that attentional modulation of auditory neuron response properties can act to improve signal-to-noise ratios and the reliability of sensory encoding, does selective attention allow subjects to focus on specific sound features in a complex auditory environment? Ideally, a subject in a complex auditory scene could utilize the spectrotemporal content of relevant features to separate attended streams from background unattended streams to better isolate the target. Task engagement has indeed been shown to result in rapid adaptions to auditory neuron response properties in a manner that optimizes encoding of task-specific features. Combined measures of temporal and frequency sensitivity to sound stimuli can be measured by calculating the spectrotemporal receptive fields (STRFs) of cortical neurons. In a series of experiments where ferrets were trained to discriminate a tonal target in the presence of background noise stimuli that were comprised of TORCs (temporally orthogonal ripple combinations), it was demonstrated that the STRFs of ACx neurons dynamically adapt to the stimulus features, enhancing responses to the target frequency while reducing responses to the non-target spectral and temporal features (Figures 2Ai,ii) (Fritz et al., 2003, 2005a). These changes in receptive properties were rapidly and specifically modulated by task-engagement, with the STRFs returning to their original fields shortly after the behavioral task was over. Moreover, STRF changes were highly dependent upon the nature of the task and revealed task-specific signatures based on whether the animal was taxed with spectral or temporal feature discrimination (Fritz et al., 2007). Task reward structure also modulates attention-driven receptive field plasticity, with positive or negative reinforcement for the same target tone resulting in rapid and selective changes in cortical STRFs at the target frequency in equal magnitude but opposite direction (David et al., 2012). Thus, attention reshapes cortical tuning properties in manner that enhances the contrast between task relevant stimulus classes. Encoding of sound spatial location is also highly sensitivity to task demands. In a task where cats were trained to identify changes in sound source origin across trials, spatial selective tuning emerged within seconds of task onset and was mediated via suppression of tuning responsivity in least preferred spatial locations (Figures 2Bi,ii) (Lee and Middlebrooks, 2011). Thus, while spatial tuning is typically broad in auditory cortical neurons, selective attention can rapidly sharpen spatial tuning properties to improve localization of attended sound sources. These findings suggest that auditory selective attention can mediate short-term cortical plasticity to modulate spectrotemporal and spatial sound encoding, thereby improving perceptual performance in a task-specific manner.

While the above studies indicate that top-down attentional signals dynamically reshape receptive fields in the primary ACx in a task-specific manner, an important question that remains is the anatomical locus of these top-down signals. As sound information ascends through the auditory system, neurons preferentially encode more abstract sound entities or categorizations rather than detailed spectrotemporal features (Chechik and Nelken, 2012; Mesgarani et al., 2014a). This abstraction along cortical hierarchies is likely important for building invariant representations of foreground target sounds that are robust to different background sound conditions. Indeed, neurons in non-primary ACx exhibit greater invariance in encoding acoustically distorted communicative signals compared to neurons in primary ACx (Carruthers et al., 2013, 2015). Similarly, dual recordings from primary and secondary ACx in ferrets trained to detect streams of repeated noise samples embedded in a stream of random background samples found that stream-specific gain enhancement was stronger in secondary cortical areas compared to primary ACx (Saderi et al., 2020). Importantly, categorical sound representations in higher-order cortical regions are often behaviorally-gated, adaptively assuming different states or filter properties depending upon the demands of the ongoing task. In ferrets engaged in a tone-discrimination task, belt regions of ACx that typically reflect stimulus properties similar to primary ACx (Figure 2Ci) take on less faithful representation of the stimulus and more abstract properties that reflect components of the motivational properties of the behavioral task (Figure 2Cii) (Atiani et al., 2014). This effect of task engagement is seen to even greater degree in frontal cortical regions, where neurons that rarely responded to sound stimuli during passive listening selectively responded to target sounds during behavior (Figure 2Ciii) (Fritz et al., 2010). These task-specific responses in frontal cortical regions could in principal provide top-down signals that mediate receptive field changes in primary ACx based on task category expectations (Fritz et al., 2007). Consistent with this notion, changes in frontal cortex representations coincide with augmented inter-areal coherence between frontal cortex and regions of primary ACx that were most responsive to target sounds (Fritz et al., 2010). Likewise, pairing electrical stimulation of orbitofrontal cortex with sound stimuli in passively listening animals induced rapid changes in the frequency receptive fields of primary ACx neurons in a manner similar to the effects of task-engagement (Winkowski et al., 2013). These findings suggest that functional interactions between frontal and primary sensory areas can shape the flow of relevant auditory information during active listening and is thus likely to play an important role in auditory scene analysis.

### Top-Down Modulation of Stimulus Statistic Adaption

Behavioral task engagement is not only associated with attention driven changes to sound feature encoding but has also been shown to directly influence the degree of bottom-up adaptation to statistical changes in sound. For instance, a recent study revealed that the magnitude of dynamic range adaptation in the IC of macaques was enhanced in animals actively engaged in a tone-in-noise detection task compared to when they were passively listening to the same stimuli (Rocchi and Ramachandran, 2020). Recordings of IC neurons in guinea pigs repeatedly exposed to a switching stimulus that alternates between loud and quiet environment found that auditory midbrain neurons adapt more rapidly with repeated exposure to a loud environment, a phenomenon termed meta-adaptation (Robinson et al., 2016). This meta-adaptation suggests that auditory scene analysis is not only influenced by the statistical properties of sound input but our prior knowledge of the sound environment. Interestingly, cortical inactivation via cryoloop cooling disrupted meta-adaptation in the IC, indicating the top-down nature of this phenomenon. Thus, adaptation to mean sound level is accelerated and more efficient when animals have been previously exposed to an environment or are engaged in an actively listening paradigm. Together, these attentional effects on spectral-temporal receptive properties, spatial tuning, and sound level adaptations are likely to aid in our ability to identify target sound sources in complex listening conditions.

### Attentional Contributions to Auditory Scene Analysis in Humans

Animal studies have clearly demonstrated that attentional state and experience can influence auditory response properties and adaption to sound stimulus statistics. Parallel human studies have provided evidence that this attention-driven plasticity is indeed important for auditory scene analysis. In human ACx, selective attention enhances psychophysical performance through increases in neural gain (Kauramäki et al., 2007; Kerlin et al., 2010; Zion Golumbic et al., 2013). Increases in multiplicative gain in ascending pathways as well as enhancement of feature selectivity in secondary auditory cortices associated with “what” and “where” processing pathways appears to occur during auditory scene analysis (Ahveninen et al., 2006). The adoption of stimulus parameters from electrophysiological studies in rodents and ferrets have further revealed complex associations in auditory regions in human studies. For instance, rapid changes in the spectrotemporal response of recorded neurons in human ACx can occur in seconds, mimicking the effects seen in ferrets. Such changes also corresponded with improved perceptual performance in extraction of speech from a degraded stimulus (Holdgraf et al., 2016). In binaural task designs, selective auditory attention enhances the neural representation of relevant sound streams while reducing the neural representation of irrelevant sound streams in distracting environments (Bidet-Caulet et al., 2007). For example, in a study with competing sound streams that were modulated at different frequencies, the auditory steady state response was modulated at the sound stream frequency in the direction of the attended stream within the precentral sulcus (Bharadwaj et al., 2014), a region important for visual spatial attention (Wardak et al., 2006). Imaging studies have further identified a dichotomy of processing where primary regions show enhanced sensitivity to temporal coherence and associate auditory regions show strong activation by sounds with reduced temporal correlations (Zatorre and Belin, 2001; Schönwiesner et al., 2005; Overath et al., 2008).

The level of noise invariance is also highly regulated by the directionality of the attended source in humans, suggesting that hierarchical cortical processing allows for spectrotemporal feature extraction that is strongly spatially modulated (Mesgarani and Chang, 2012; Schneider and Woolley, 2013). High-density EEG has revealed spatial speech stream segregation occurs during selective attention for an attended talker. Importantly, differences in alpha power (8–12 Hz) across hemispheres at parietal sites indicated the direction of auditory attention (Kerlin et al., 2010). Interestingly, analysis of high gamma (75–150 Hz) LFPs in the posterior temporal lobe reveals that reconstruction of the speech spectrograms from neural activity reflect the attended speaker alone despite being presented in the presence of a competing speaker. Importantly, on counterbalanced trials, the reconstructed spectrograms in the same region reflected the change to the new attended speaker, suggesting cortical representation of speech gives rise to the perceptual aspects relevant for the listener’s intended goal (Mesgarani and Chang, 2012). In subsequent studies, Deng et al., found that directed attention cues occurring before the auditory discrimination task promoted supramodal alpha activity ipsilateral to the area of directed attention. Further, this relative ratio of ipsilateral/contralateral alpha activity shifted smoothly across hemispheres as the target source location was moved from the ipsilateral to the contralateral location (Deng et al., 2020). Such findings suggest that an ability to attend to localized sound statistics reflecting a relevant target are an important feature of auditory scene analysis, although not all listeners can do this with the same level of precision. For instance, a recent study in individuals with normal levels of hearing and speech understanding, found that reduced performance for non-speech auditory selective attention accounted for the greatest variation in individual task performance in a cocktail-party listening task (Oberfeld and Klöckner-Nowotny, 2016). This raises the intriguing possibility that listener performance in complex environments is largely a function of attentional capacity. These findings offer insight into the complex interactions between sound feature statistic adaptation and the role of cognitive capacity in attentional gain control during auditory scene analysis.

This section has highlighted our current understanding of behavioral state influences on auditory processing and the evidence for top-down regulation of feature encoding and adaptation in the auditory system. However, several questions remain to be answered. For instance, despite strong evidence for attentional gain control and task-dependent receptive field plasticity in the auditory system, it is still unclear if gain modulation can sufficiently account for the receptive field changes seen with task engagement (Otazu et al., 2009; Lopez Espejo et al., 2019). In addition, while frontal executive control regions are implicated in top-down modulation of auditory feature encoding through studies of coherence, the specific cortical regions involved— and how they are recruited to impart influence in primary sensory regions— remains unclear. Most prominently, little is known about the local and long-range circuit mechanisms and neurotransmitter systems that allow for such dynamic attentional adaptations. In the subsequent section we will discuss potential candidates, including distinct neuronal subtypes which confer specialization to cortical and subcortical circuits during sensory processing.

## Mechanisms for Bottom-Up and Top-Down Adaptations

**FIGURE 3:**
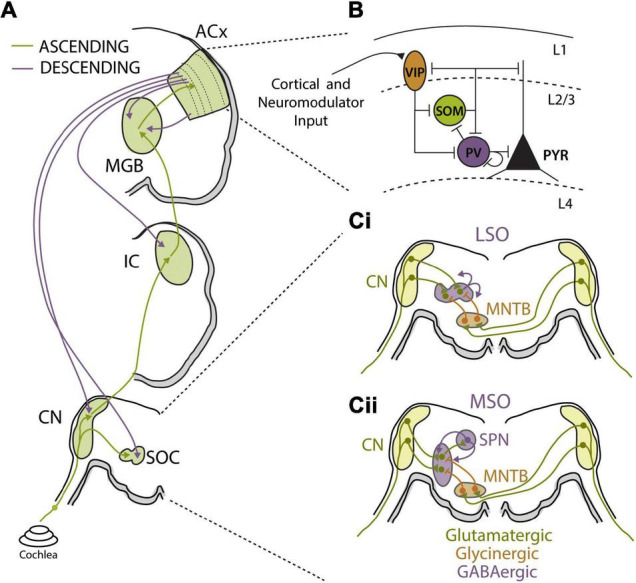
Auditory circuit mechanisms for bottom-up and top-down adaptations. **(A)** Schematics of major auditory ascending (green) and descending (purple) pathways and associated auditory processing nuclei from the cochlea to auditory cortex (ACx). Major sound processing nuclei are highlighted in green, including the cochlear nucleus (CN), superior olive complex (SOC), inferior colliculus (IC), medial geniculate body (MGB), and ACx. Ascending pathways primarily terminate in layer 4 of ACx while corticofugal projections originate in layer 5b and layer 6 and can terminate at every level of the ascending pathway. **(B)** Summary figure showing known inhibitory relationships between parvalbumin (PV), somatostatin (SST), and vasoactive intestinal peptide (VIP)-positive neurons and their combined influences on excitatory cell populations. In this largely accepted model, locomotion, or top-down input, preferentially activates VIP cells, reducing SST cell output and releasing PV and excitatory cells from inhibition. PV activity can reduce background noise that improves signal-to-noise encoding of sensory stimuli by excitatory cell populations. Cortical layers are shown and separated by dashed lines. Figure was adapted from Pakan et al. (2016). **(Ci,ii)** Schematics of sound localization circuits of auditory brainstem used for processing interaural level **(Ci)** and timing **(Cii)** differences. **(Ci)** Principal neurons of lateral superior olive (LSO) receive excitatory glutamatergic inputs (green) from ipsilateral CN and glycinergic inhibition (orange) from ipsilateral medial nucleus for the trapezoid body (MNTB). MNTB receives excitatory input from contralateral CN. The interaction of ipsilateral excitation and contralaterally driven inhibition drives LSO firing in manner that can be used to calculate interaural level differences (ILD). LSO principal neurons release GABA (purple) in activity-dependent manner to asymmetrically modulate function of both glutamatergic and glycinergic inputs via activation of pre-synaptic GABA_*B*_ receptors. **(Cii)** Principal neurons of the medial superior olive (MSO) receive excitatory glutamatergic inputs (green) from ipsilateral CN and contralaterally driven glycinergic inhibition (orange) from ipsilateral MNTB. MSO neurons also receive ipsilaterally driven inhibition from lateral nucleus of the trapezoid body (not shown). MSO neurons send excitatory projections to superior olivary nuclei (SPN), which in turn send feedback GABAergic projections (purple) to the MSO.

The combination of bottom-up adaptations to the sound statistical environment and top-down modulation of receptive field properties greatly effects sound feature encoding in the auditory system and is likely to impact our ability to listen to target sound sources in a noisy environment. There is also evidence that these bottom-up and top-down adaptations directly interact, as task engagement can modulate how auditory neurons adapt to changes in incoming stimulus statistics. An important question, therefore, is if these bottom-up and top-down adaptations converge on common neuronal mechanisms. Does attention co-opt the circuits that mediate bottom-up gain adaptations, or do top-down and bottom-up gain control rely on independent mechanisms that can interact in complex ways? There are number of cellular and circuit mechanisms that may be used to implement gain adaptations to changes in incoming stimulus statistics and/or selective attention (Figure 3). This section will discuss our current understanding of how these changes are implemented within the auditory system and potential interactions between them.

### Synaptic Mechanisms Contributing to Sound Stimulus Adaptations

Efficient information processing in neural circuits is dependent on tightly regulated interactions between excitatory and inhibitory neurons, which may or may not necessarily be balanced depending on conditions or behavioral state (Haider et al., 2006; Shew et al., 2011; Yizhar et al., 2011). Under conditions of tight excitatory-inhibitory balance (E/I balance), as sometimes seen in the ACx (Wehr and Zador, 2003; Zhang et al., 2003), synaptically driven fluctuations in membrane potential (*V*_*m*_) can multiplicatively regulate tuned neural responses (Chance et al., 2002; Shu et al., 2003). Thus, noisy background synaptic input can exert gain control on neuronal output under different environmental conditions, providing a potential cellular mechanism for gain adaptations to stimulus statistics (Finn et al., 2007). However, *in vivo* whole cell recordings from ACx neurons during high and low contrast stimulation found that membrane conductance was not significantly modulated by stimulus contrast and contrast-dependent *V*_*m*_ fluctuations could not account for contrast gain control in the ACx (Cooke et al., 2020). Short-term synaptic plasticity is another potential mechanism for gain modulation (Abbott et al., 1997), as short-term synaptic depression at thalamocortical synapses is believed to contribute to contrast gain control in the visual system (Carandini et al., 2002; Banitt et al., 2007). Synaptic depression is observed at synapses across levels of the auditory system (Nelson et al., 2009; Yang and Xu-Friedman, 2009; Blundon et al., 2011), including hair cell-ribbon synapses (Goutman, 2017). While this form of short-term plasticity has been proposed to play a role in SSA (Motanis et al., 2018), its role in stimulus statistic adaptation remains to be determined. Interestingly, STRF models that incorporate both synaptic depression and gain control show that there are additive effects of these properties on the robustness of cortical neuron STRFs to background noise, suggesting that these are complementary processes that may not reflect the same underlying mechanism (Mesgarani et al., 2014b; Pennington and David, 2020).

Modulation of presynaptic synaptic transmission has been shown to play an integral role for adaptive gain control in auditory brainstem circuits that use precise comparison of excitatory and inhibitory synaptic inputs to compute ITDs and ILDs for sound localization (Figures 3Ci,ii) (Finlayson and Caspary, 1989; Park et al., 1996). Interestingly, both excitatory and inhibitory pre-synaptic terminals in the MSO and LSO are dynamically adjusted by GABA via activation of pre-synaptic GABA_*B*_ receptors that modulate neurotransmitter release (Magnusson et al., 2008; Grothe and Koch, 2011; Stange et al., 2013). In the LSO, GABA is released from principle cells in an activity-dependent manner and bind to pre-synaptic GABA_*B*_ receptors to mediated gain adaptation on the time scale of seconds (Figure 3Ci) (Magnusson et al., 2008). Retrograde activation of presynaptic GABA_*B*_ receptors has asymmetric effects on excitatory and inhibitory synaptic terminals in the LSO, suppressing glutamatergic transmission more strongly than glycinergic transmission. The net effect is to decrease excitability of LSO neurons, resulting in a shift in the dynamic range of ILD functions and narrowing the binaural receptive field of LSO neurons so that ipsilateral stimuli are preferentially encoded and perceived as more intense (Magnusson et al., 2008). MSO neurons have also been shown to modulate their sensitivity to ITD through a GABA_*B*_ feedback mechanism from the superior periolivary nucleus (SPN), which also receives collateral inputs from the MSO (Figure 3Cii). This di-synaptic feedback loop activates pre-synaptic GABA_*B*_ receptors, causing a slow-acting and long-lasting decrease in MSO neuronal activity in a manner proportional to their prior activity levels. This activity-dependent rate adaptation does not directly alter preferred ITDs in MSO neurons, but results in a form output normalization gain modulation that produces asymmetry in hemispheric population code for sound space (Stange et al., 2013). In this manner, strongly lateralized sound sources induce unequal adaptation preferentially in the contralateral hemisphere, thereby shifting perceived location of a subsequently presented sound source. Parallel psychophysical experiments found that the same paradigm used to evoke GABA_*B*_ receptor-mediated adaptation in gerbils caused predictable shifts in sound localization percepts in humans (Phillips and Hall, 2005; Stange et al., 2013). Thus, dynamic adjustments to the balance between excitation and inhibition in MSO and LSO neurons via regulation of presynaptic transmitter release are used to modulate sound localization cues and spatial perception.

### Cortical Circuit Mechanisms Contributing to Bottom-Up and Top-Down Adaptations

Local inhibitory interneuron networks are prominent regulators of neuronal gain, particularly within cortical circuits (Katzner et al., 2011; Ferguson and Cardin, 2020), and E/I balance is thought to be essential for proper regulation of sensory encoding (Wehr and Zador, 2003; Isaacson and Scanziani, 2011). Indeed, there is evidence that alterations to E/I balance underlie rapid receptive field changes seen in cortical neurons following experimental conditioning or learning (Carcea and Froemke, 2013; Froemke et al., 2013). E/I balance is also highly state-dependent (Haider et al., 2013; Zhou et al., 2014) and top-down modulation of E/I balance may contribute to the observed behavioral state and attentional gain modulation of sensory processing discussed in the previous section (Harris and Thiele, 2011). However, the diversity of inhibitory interneurons subtypes, in terms of physiological properties and computational functions, has complicated our understanding of how local inhibitory networks contribute to sensory gain adaptations. Most recent work has focused on three major GABAergic cell types (Figure 3B): Fast-spiking parvalbumin positive (PV) interneurons that target perisomatic regions of excitatory neurons; low-threshold spiking somatostatin positive (SST) interneurons that target dendrites; and sparse, dendritic targeting vasoactive intestinal peptide (VIP) interneurons that often target other inhibitory interneurons to form disinhibitory circuits (Wood et al., 2017).

PV interneurons act as key mediators of response gain in cortical principal cells (Cardin et al., 2009; Sohal et al., 2009; Atallah et al., 2012; Wilson et al., 2012) and have been broadly implicated in feedforward circuits contributing to frequency tuning, adaptation, and gap encoding in ACx (Moore and Wehr, 2013; Li et al., 2014, 2015; Aizenberg et al., 2015; Natan et al., 2015; Keller et al., 2018). For these reasons, recent work has attempted to elucidate the role of PV neurons in contrast gain control in the ACx (Cooke et al., 2020). Optogenetic manipulation of PV neurons did indeed modulate the overall gain of ACx principal neurons. However, PV-mediated inhibition was minimally involved in gain adaptations to changes in sound variance and PV interneuron activity itself was not modulated by stimulus contrast. Thus, PV neuron activity modulates the gain of auditory cortical responses but not in a contrast-specific manner. Consistent with these findings, both background noise and PV neuron activation alter response gain of ACx principle neurons but the effects of these manipulations are additive, suggesting they involve independent mechanisms (Christensen et al., 2019). It is important to note, however, that similar levels of contrast gain adaptation are observed in the cortex and subcortical structures in mice (Lohse et al., 2020). The fact that local manipulation of PV neurons in the ACx does not influence contrast gain modulation does not preclude a role for inhibitory circuits in subcortical auditory structures. It will be important to use similar approaches to examine the role of inhibition in subcortical auditory areas as well as the role of other inhibitory interneuron cell types in the cortex.

On the other hand, recent work has demonstrated that PV neuron activity in the ACx is strongly modulated by behavioral state, suggesting that this class of interneurons may be involved in top-down attentional gain modulation. *In vivo* whole-cell recordings in awake mice found that spontaneous and sensory-evoked responses of both excitatory and PV neurons in the ACx are scaled down when animals transitioned from quiescence to active behavior (Zhou et al., 2014). This behavioral-state dependent gain modulation preserved tuning properties of ACx principle neurons but increased signal-to-noise ratios by relatively suppressing spontaneous activity more than evoked activity. PV neurons are also strongly regulated by motor cortical projections that act to suppress ACx activity associated with internally generated acoustic stimuli during locomotion (Nelson et al., 2013; Schneider et al., 2014). While it remains to be determined how attention engages PV neurons in the auditory system, PV neuron activity in the prefrontal cortex is increased with goal-driven attentional processing and PV neuron activity levels correlated with behavioral performance on the 5-choice serial reaction time task, a common rodent attentional task (Kim et al., 2016). Importantly, PV neurons play in integral role in generation of gamma oscillations in the cortex (Cardin et al., 2009; Sohal et al., 2009) and attentional processing is characterized by increases in gamma activity in sensory regions (Fries et al., 2001; Gregoriou et al., 2015; Ni et al., 2016). Gamma activity has been suggested to modulate the gain of incoming sensory input (Tiesinga et al., 2004, 2008; Börgers et al., 2005; Ni et al., 2016), providing a link between PV neuron function and attentional gain control (Tiesinga et al., 2004, 2008; Börgers et al., 2005; Ni et al., 2016). Thus, while PV neurons in the cortex may not be necessary for bottom-up contrast gain control, they are likely to be important mediators of top-down attentional gain modulation.

SST interneurons have been implicated in a variety of forms of auditory cortical adaptations, including SSA (Kato et al., 2015; Natan et al., 2015, 2017) and forward suppression (Phillips et al., 2017). While a role for SST neurons in contrast gain control or other forms of stimulus statistic adaptation remain to be determined, cortical SST neurons do exhibit properties that make them well-suited for these types of computations compared to PV neurons. For instance, while PV neurons are co-tuned for frequency with neighboring excitatory neurons in the ACx (Moore and Wehr, 2013), SST neurons are involved in a form of network-level lateral inhibition in the cortex (Kato et al., 2017). This lateral inhibitory network could provide a substrate for divisive normalization, a canonical computational strategy used throughout sensory systems to implement gain modulation for invariant sensory representation (Olsen and Wilson, 2008; Carandini and Heeger, 2012). SST neurons are also recruited slightly later than PV or excitatory cells, and SST neurons are more tightly tuned with higher intensity thresholds, suggesting they may contribute to feedback modulation of cortical circuits in response to stimulus history (Li et al., 2014, 2015; Kato et al., 2017).

A third important cortical inhibitory cell-type is the VIP expressing interneuron. VIP neurons represent only 1–2% of cortical neurons but can have broad impact on cortical circuit function, as they target other cortical interneurons in superficial layers (Figure 3B) (Pfeffer et al., 2013). VIP neurons also receive strong neuromodulator input and are highly innervated by intracortical projections from outside of primary sensory areas (Zhang et al., 2014). These properties make VIP neurons well-suited to implement top-down modulation of cortical response gain via disinhibition. Consistent with this notion, VIP neuron activity in visual cortex is upregulated during locomotion and optogenetic activation of VIP neurons increases response gain of visual cortical excitatory neurons, mimicking the effect of locomotion (Fu et al., 2014). Optogenetic stimulation of VIP interneurons during a visual contrast detection task improves performance, while activating either SST or PV interneurons reduces the ability of the mouse to detect lower contrasts (Cone et al., 2019). Similar effects are seen for frequency tuning in the ACx, where VIP activation transiently suppresses SST and, to a lesser extent, PV neuron activity, leading to disinhibition in a subset of tone-responsive neurons and an increase in the gain of the corresponding tuning curves (Pi et al., 2013). Moreover, this study demonstrated that VIP neurons were strongly recruited in response to reinforcement signals during a tone discrimination task. Thus, VIP neurons are particularly well-suited to mediate top-down gain modulations via a disinhibitory cortical microcircuit that is engaged under specific behavioral conditions, and may therefore play an important role in attentional modulation of auditory processing.

### Cortico-Fugal Circuits Contributing to Bottom-Up and Top-Down Adaptations

Descending projections from the ACx are far more numerous than ascending projections, and these massive yet poorly understood corticofugal projections target virtually every level of the auditory system, including the MGB, IC, cochlear nucleus (CN), superior olivary complex (SOC) and even the cochlea (Figure 3A) (Winer et al., 2002; Xiao and Suga, 2002; Meltzer and Ryugo, 2006; Llano and Sherman, 2009; Jäger and Kössl, 2016). While we are only beginning to understand how these descending projections influence sound perception, there is strong evidence for top-down regulation of subcortical sound processing via corticofugal projections. As with local cortical inhibitory neurons, corticofugal neurons are a heterogeneous set of cells with diverse properties and projection targets. Early studies revealed that stimulating cortico-thalamic (CT) projecting fibers egocentrically enhances tuning to match the origin of the descending cortical region (Yan and Suga, 1996; Zhang et al., 1997). More recent work has identified the complexity of this pathway in serving to balance the competing demands of increasing neuronal sensitivity for rapid signal detection or dampening excitability to enhance fine-tuned feature discrimination (Happel et al., 2014; Guo et al., 2017; Homma et al., 2017). While activation of CT neurons has been shown to decrease cortical response gain via direct activation of local inhibitory interneurons and/or projections to the TRN (Olsen et al., 2012; Bortone et al., 2014), recent evidence indicates that CT neurons can drive both increases and decreases to cortical gain depending on the timing of their activation relative to ascending input (Crandall et al., 2015; Guo et al., 2017). Cortico-collicular (CC) projecting neurons make-up a distinct class of corticofugal neurons with different projection targets and response properties than CT neurons (Winer et al., 2002; Williamson and Polley, 2019). CC projections to the IC enhance SSA and sharpen frequency tuning, biasing the receptive fields of subcortical auditory neurons toward frequently occurring or highly salient stimuli (Zhang et al., 2005; Bajo et al., 2010; Blackwell et al., 2016).

How do descending auditory projections contribute to stimulus statistic adaptation? While cortical silencing has significant effects on neuronal excitability in the MGB and IC, this manipulation does not affect contrast gain control (Lohse et al., 2020) or mean level adaptation (Robinson et al., 2016) in these structures, indicating that subcortical gain and dynamic range adaptations occur independently of top-down cortical feedback. However, cortical inactivation did interfere with meta-adaptation in the IC (Robinson et al., 2016) and several studies have shown that auditory attentional tasks modulate efferent projections back to the cochlea (Giard et al., 1994; Marian et al., 2018). Thus, it has been proposed that corticofugal projections play an important role in providing contextual information to upstream auditory areas that is necessary for interpreting ambiguous signals, such as those encountered in complex or noisy acoustic environments (Asilador and Llano, 2021). Indeed, electrical stimulation of ACx in humans was shown to modulate subcortical auditory pathways and enhance speech recognition under challenging conditions (de Boer and Thornton, 2008; Srinivasan et al., 2012; Shastri et al., 2014). Together, these studies suggest that descending corticofugal projections likely play an important role in top-down modulation of auditory gain in response to changes in behavioral state or context but may not be necessary for adaption to sound statistics observed in subcortical auditory areas.

Within this section we have provided a summary of our current understanding of cellular and circuit mechanisms that contribute to bottom-up and top-down adaptation throughout the auditory system. We have focused on both local synaptic and circuit interactions between excitatory and inhibitory neurons as well long-range connections between auditory regions that play essentials roles in adaptive sound processing. The role of specific interneuron subclasses and their specialized contributions to subcortical and cortical microcircuits remains an active area of interest in sensory processing. In particular, the contribution of specific interneuron classes to bottom-up stimulus statistic adaptation and top-down task-dependent receptive-field plasticity remains to be fully elucidated. While recent work has indicated that PV neurons are unlikely to mediate contrast gain control in the ACx, the role of other interneuron subtypes is less well understood. Recent computational studies have suggested that top-down inhibitory neurons that disinhibit bottom-up cortical circuits, similar to the VIP neuron circuit motif described above, can explain the attentional effects of auditory tuning properties (Chou and Sen, 2021). However, this model remains to be tested experimentally. Furthermore, much less is known about the role of interneurons subcortically as it relates to whether the behavioral state modulations that are actively present in cortex contribute to adaptation and meta-adaptation observed in ascending subcortical structures. Finally, a major challenge for the field going forward is how to best isolate the contributions of these cell classes due to their interconnected nature and the presence of disinhibitory effects that are difficult to disentangle while using traditional methods of manipulation.

## Hearing Loss and Hearing in Noisy Environments

One of most prominent and disabling disruptions associated with hearing loss is the inability to hear in noisy environments. Difficulties hearing in noise could be due to a general reduction in audibility and degraded encoding of incoming sound input as a consequence of hearing loss. However, studies have shown that speech perception and hearing in noise difficulties are present even when cochlear amplification is accounted for Peters et al. (1998); Johannesen et al. (2016). Moreover, hearing in noise difficulties often occur even in the absence of overt audiometric threshold shifts (Kraus et al., 2000; Zeng et al., 2005). Hearing loss fundamentally alters the pattern and level of incoming sound, and thus will greatly affect the stimulus statistics the auditory system is exposed to. Indeed, hearing loss is often associated with central auditory gain enhancement in attempts to preserve sound detection levels (Auerbach et al., 2014; Chambers et al., 2016; Salvi et al., 2016). Thus, it is possible that compensatory gain changes with pathological changes to auditory input and adaptations to more physiological changes to sound statistics engage overlapping mechanisms and may interfere with each other. Hearing loss may also interfere with attentional mechanisms important for sound perception. Selective attention performs best when auditory streams can be segregated based on select features. Degradation of spectrotemporal structure impairs adaptation accuracy and reduces the efficiency of anticipated noise. Inaccuracies build at multiple levels, delaying and reducing the efficiency with which attention groups relevant objects. In complex scenes, where background noise statistics and the spectrotemporal features of the target can rapidly fluctuate, hearing impaired listeners have more difficulty forming perceptual objects from their environments (Shinn-Cunningham and Best, 2008). Under these conditions, the benefits of knowing what features to direct attention to are degraded and reduce the capacity for cognitive control to benefit the listener. Thus, hearing loss not only affects bottom-up gain adaptations, but these changes are compounded by reducing the capacity for top-down attentional mechanisms to help contribute to auditory scene analysis. In the preceding sections, we have reviewed the primary bottom-up and top-down adaptations that contribute to auditory scene analysis, as well as the potential mechanisms underlying their generation. In this section we will discuss how our understanding of these adaptive coding strategies in normal hearing can help us understand the often devastating effects of hearing loss on listening in complex acoustic environments.

### Hidden Hearing Loss

**FIGURE 4:**
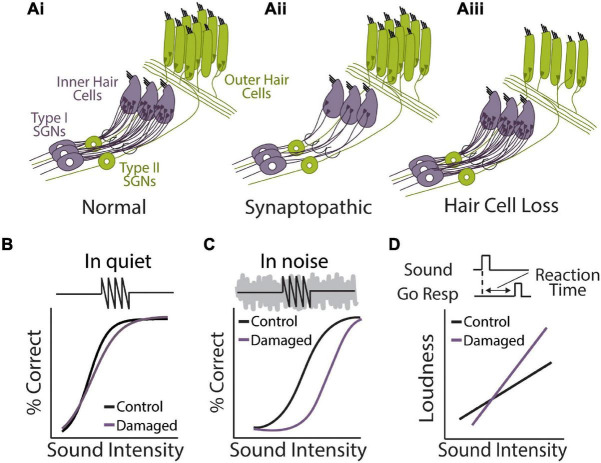
Perceptual consequences of sensorineural hearing loss. **(Ai,ii,iii)** Diagram of cochlear hair cells and spiral ganglion neuron connectivity under normal **(Ai)** or pathological conditions of synaptopathy **(Aii)**, or sensory hair cell damage **(Aiii)**. **(Ai)** Purple: over 95% of afferent input to central auditory system comes from type 1 spiral ganglion neurons (SGN) that form synaptic contacts inner hair cells (IHCs), the main conventional sensory receptors of the cochlea. IHCs are innervated by multiple (10–20) type I neurons but each type I neuron only contacts a single IHC. Green: unmyelinated type II SGNs form synaptic contacts with multiple outer hair cells (OHCs) but each OHC only receives one contact from one Type-II neuron. Type-II SGNs represent only 5% of afferent input and are not involved with transmission of acoustic information to brain. Rather, the major role of OHCs is to amplify the cochlear mechanical response to low-level input, providing increased sensitivity to low intensity sounds. **(Aii)** In the synaptopathic ear, many of the synaptic contacts between type I SGNs and IHCs have degenerated, leaving fewer afferent nerve fibers to relay sound information from the ear to the brain, which may underly hidden hearing loss and impaired speech-in-noise perception. **(Aiii)** Many forms of acquired sensorineural hearing loss are associated with damage to OHCs and disruption to mechanical cochlear gain control mechanisms, leading to permanent threshold shifts, loudness recruitment, and broader frequency tuning. **(B)** Tone detection behavior in animals with selective damage the type I SGN-IHC complex (purple) is remarkably normal under quiet conditions, even with moderate to severe cochlear deafferentation. **(C)** Tone-in-noise detection is more severely impaired in animals with selective damage the type I SGN-IHC complex (purple) even though thresholds in quiet are maintained. Schematized data in panels **(B,C)** adapted from Resnik and Polley (2021). **(D)** Auditory reaction time (RT) measures of loudness growth in animal models (Radziwon and Salvi, 2020) have demonstrated that some forms of hearing loss can result in abnormal increases to the slope of RT-intensity functions, consistent with loudness recruitment and/or hyperacusis.

Sensorineural hearing loss is often associated with overt damage to sensory hair cells, resulting in elevated sound detection thresholds (Figures 4Ai,ii,iii) (Schmiedt, 1984; Cunningham and Tucci, 2017). Permanent threshold shifts in the clinical pure tone audiogram have thus traditionally been a key criterion for diagnosing hearing loss (Simel et al., 2016). However, many individuals with clinically normal audiometric thresholds nonetheless report significant auditory perceptual disruptions, including temporal processing deficits, impaired speech perception, and most prominently, difficulties hearing in noisy environments (Starr et al., 1996; Zeng et al., 2005; Cunningham and Tucci, 2017; Ralli et al., 2019). Recent evidence from animal models has suggested that cochlear neuronal degeneration can occur even without overt hair cell damage or permanent threshold shifts (Kujawa and Liberman, 2009). This so called “hidden hearing loss” (HHL), due to the fact that this dysfunction is not revealed by standard audiometric tests, is estimated to occur in ∼12–15% of individuals (Tremblay et al., 2015; Spankovich et al., 2018). HHL is likely a key contributor to difficulties hearing in noise in the absence of clinically diagnosed hearing loss (Plack et al., 2014; Ralli et al., 2019) and has also been suggested to contribute to auditory perceptual disorders like tinnitus and hyperacusis that are often associated with hearing impairment (Schaette and McAlpine, 2011; Hickox and Liberman, 2014). Below we discuss the consequences of cochlear degeneration on peripheral and central auditory function, with a particular focus on how central adaptations to this form of hearing loss may interfere with our ability to compensate for noisy environments.

### Cochlear Synaptopathy

While there are three times as many outer hair cells (OHC) than inner hair cells (IHC), virtually all (∼95%) afferent signals from the cochlea are relayed to the central auditory system via IHCs synapsing on type 1 spiral ganglion neurons (SGNs) (Figure 4Ai) (Spoendlin, 1972). Accumulating evidence suggests that the synapses between IHCs and type I SGNs, whose axons comprise the AN tract, appear to be most vulnerable to noise- or age-related hearing loss. Indeed, animal studies have found that there is a marked reduction of IHC-Type 1 SGN synaptic contacts following exposure to ototoxic drugs, environmental noise, or aging, and this synaptopathy often proceeds overt hair cell damage (Figure 4Aii) (Liberman and Kujawa, 2017; Wu et al., 2019; Kohrman et al., 2020). Remarkably, it has been shown that animals with damage restricted to the IHC-type I SGN complex maintain normal hearing thresholds in quiet despite severely reduced afferent drive to the central auditory system (Figure 4B) (Lobarinas et al., 2013; Chambers et al., 2016). However, these same animals perform much poorer than control animals when challenged with a more difficult task, such as tone detection in background noise, gap-in-noise detection, or a remote masking paradigm (Figure 4C) (Salvi et al., 2016; Lobarinas et al., 2020; Resnik and Polley, 2021). These results indicate that cochlear degeneration could contribute to real-world listening difficulties even in the absence of threshold shifts in the clinical audiogram. It should be noted that AN fiber loss following kainic acid treatment in budgerigars, a common avian model of hearing (Dooling and Popper, 2000), does not result in deficits to tone-in-noise detection (Henry and Abrams, 2021), suggesting there may be species-specific effects of cochlear degeneration and/or central adaptation to hearing loss.

The difference in detection between quiet and noisy conditions following cochlear degeneration may be due in part to peripheral mechanisms. Spared AN fibers maintain normal thresholds and tuning following IHC or SGN degeneration and detecting tones in quiet may only require a small fraction of surviving peripheral afferents (Wang et al., 1997; Kujawa and Liberman, 2009; Salvi et al., 2016). Interestingly, the AN fibers most susceptible to noise-induced synaptopathy are low and medium SR fibers, which have higher thresholds and are thought to be useful for hearing in noisy environments (Wang et al., 1997; Furman et al., 2013). However, substantial recovery of sound detection thresholds is seen even with ototoxic treatments that cause near complete loss (∼95%) of IHC-SGN synapses (Chambers et al., 2016) or selective lesion of IHCs, which are similarly contacted by all subsets of AN fibers (Lobarinas et al., 2013; Salvi et al., 2016). In fact, IHC lesions were actually shown to result in an increased proportion of low SR nerves relative to medium and high, opposite to what is observed with noise-induced synaptopathy (Salvi et al., 2016). Thus, while peripheral mechanisms certainly contribute to perceptual alterations associated with cochlear degeneration, there is growing awareness that adaptations in the central auditory system are essential for fully understanding the perceptual consequences of cochlear hearing impairment.

### Central Gain Enhancement Following Hearing Loss

**FIGURE 5:**
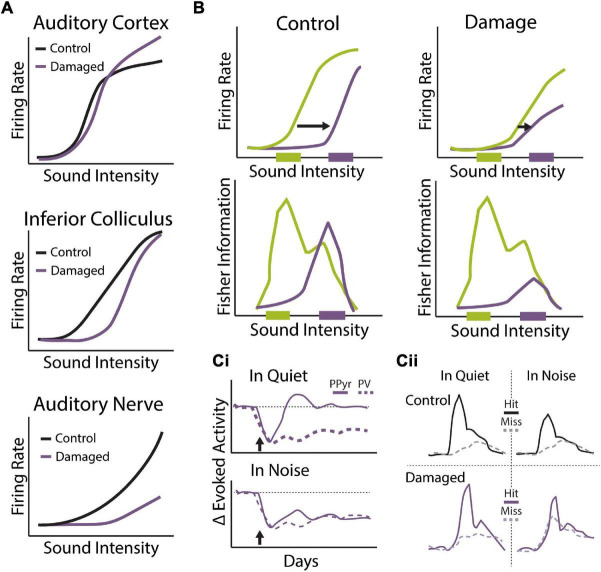
Central gain enhancement following sensorineural hearing loss. **(A)** Schematics of rate-intensity functions from multiple levels of the auditory system under control conditions (black) or following cochlear damage via noise or ototoxic drug exposure that results in the central gain enhancement (purple). While output from the AN is severely degraded in terms of evoked-response threshold and suprathreshold intensity coding, rate-intensity functions gradually recover at ascending levels of the auditory system so that thresholds and suprathreshold responses are nearly normal at the level of the ACx and, in some cases, exhibit rebound hyperactivity. **(B)** Mean sound level adaptation to loud sound environments is altered with noise-induced hidden hearing loss. Rate-intensity functions from the IC of control (left) and noise exposed (right) mice when exposed to dynamic sound stimulus that switches between distributions with high probability of low sound levels (green) and high probability of high sound levels (purple). Noise exposed animals exhibit less dynamic range adaptation (top) and response functions carry less information about loud sound environments (bottom) compared to control animals. Schematized data adapted from Bakay et al. (2018). **(Ci,ii)** Cochlear degeneration triggers compensatory changes to cortical excitatory/inhibitory balance that differentially effects tone detection in quiet and noise. **(Ci)** Distinct changes to tone-evoked calcium transients in putative excitatory pyramidal neurons (PPyr, solid lines) and genetically-labeled PV inhibitory neurons (dashed lines) in the ACx following ouabain induced cochlear degeneration (arrow). Following transient loss of evoked activity, PPyr neurons exhibit near complete recovery of evoked-response size in quiet but not in background noise. Sustained decreases to tone-evoked activity in PV neurons are observed following ouabain treatment in both quiet and noise conditions. **(Cii)** Combined behavioral and imaging sessions showing differences in tone-evoked responses in cortical PPyr neurons on hit (solid lines) vs. miss (dashed lines) trials in quiet or background noise from animals before (control, black) and after ouabain-induced cochlear degeneration (damaged, purple). Decreased tone-in-noise detection in ouabain-treated animals is not only associated with diminished tone-evoked responses on hit trials but also increased activity on miss trials. These results suggest that altered E/I balance in the ACx following cochlear degeneration may lead to impaired adaption to background noise and decreased signal-to-noise ratios for detection of foreground stimuli. Schematized data adapted from Resnik and Polley (2021).

Loss of afferent drive to the central auditory system— be it due to ototoxic drugs, sensorineural hearing loss, or acoustic deprivation— have been shown to result in a compensatory increase in neuronal gain in the central auditory system, a phenomenon termed central gain enhancement (Figure 5A) (Gerken et al., 1984; Syka, 2002; Auerbach et al., 2014; Chambers et al., 2016; Salvi et al., 2016). Gain increases due to sensorineural hearing loss have been observed at every level of the auditory system. AN synapses onto CN bushy cells have been shown to homeostatically adapt their pre-synaptic strength in response to changes in acoustic input (Zhuang et al., 2020), indicating that dynamic gain adaptations are in place at the earliest points of the central auditory system. Indeed, neuronal gain increases in response to hearing loss have been observed in several auditory brainstem nuclei, albeit in a cell-type specific and time-restricted manner (Boettcher and Salvi, 1993; Brozoski et al., 2002; Cai et al., 2009). However, in studies where concurrent recordings from multiple levels of the auditory system were performed, it has been consistently found that gain changes with drug or noise-induced hearing loss are more rapid and more complete in the ACx compared to subcortical structures like in the IC or CN (Figure 5A) (Popelar et al., 1987; Syka et al., 1994; Auerbach et al., 2014; Chambers et al., 2016; Salvi et al., 2016). Thus, like more rapid adaptations to stimulus statistics, sustained gain increases following pathological changes to sound input progressively develop through the ascending auditory system, with the most complete recovery being observed at the level of the ACx (Figure 5A). Central gain enhancement following cochlear degeneration has also been shown to result in enhanced sound-evoked activity in corticofugal projections, suggesting that in addition to changes along the ascending auditory pathway, descending projections are also altered in response to hearing loss (Asokan et al., 2018).

What are the perceptual consequences of central gain enhancement? There is growing evidence that central gain enhancement is associated with restoration of hearing thresholds in quiet (Figure 4B). Parallel behavioral and neurophysiological studies in mice given round window application of the Na^+^/K^+^ ATPase pump inhibitor ouabain, which selectively destroys type-I SGNs, or chinchillas treated with the anti-cancer agent carboplatin, which selectively destroys IHCs in these animals, have shown that recovery in tone detection thresholds in quiet (Figure 4B) corresponds with recovery of intensity-response functions in ACx (Figure 5A) (Chambers et al., 2016; Salvi et al., 2016). However, this compensatory plasticity is unable to restore all aspects of auditory processing that are disrupted by sensorineural hearing loss and, in fact, may actively contribute a range of auditory perceptual deficits associated with hearing impairment as well. For instance, while central gain enhancement restores mean firing rates and intensity coding in the ACx, this adaptation cannot compensate for degradation of temporal processing with hearing loss, which depends on specialized subcortical circuits optimized for fast time scales (Chambers et al., 2016). Interestingly, gain increases in the ACx following hearing loss can often overshoot baseline levels, resulting in sound-evoked hyperactivity (Figure 5A). This excessive increase in central gain may contribute to the development of hyperacusis, a sound intolerance disorder often associated with hearing loss (Zeng, 2013; Auerbach et al., 2014; Pienkowski et al., 2014). Indeed, cortical gain increases are associated with maladaptive changes to loudness perception following ototoxic (Auerbach et al., 2018) or noise-induced hearing loss (Figure 4D) (Radziwon et al., 2019). These results highlight the perceptual trade-offs that inevitably arise when sensory systems must adapt their neural representations to changes in the environment.

Recent evidence suggests the intriguing notion that central gain changes that support restoration of sound processing in quiet backgrounds may actively interfere with auditory circuit mechanisms that normally support adaptation to background noise. While thresholds in quiet are remarkably normal in animals with severe cochlear degeneration, performance in hearing damaged animals was much worse when challenged with a tone-in-noise detection task (Figure 4C) (Lobarinas et al., 2013; Salvi et al., 2016; Resnik and Polley, 2021). Examination of mean-level adaptation in the IC of mice given a noise exposure that produces HHL and central gain increases found significant impairment in adaptive coding for loud environments (Bakay et al., 2018). While both dynamic range and gain adaptations were still observed in the IC of noise-exposed animals, threshold shifts were significantly reduced compared to controls and intensity-response functions were less informative about the sound level distribution the animals were exposed to, particularly for loud environments (Figure 5B). This impairment in adaptive coding could contribute to difficulties hearing in noisy environment. Indeed, a follow-up study found that noise-induced HHL in gerbils, which have a hearing range more similar to humans than mice, reduced the ability of IC neurons to discriminate between speech tokens presented in background noise at high sound intensities (75 dB SPL), although discriminability at moderate sound intensities (60 dB SPL) was surprisingly improved (Monaghan et al., 2020). A phenomenological model of cochlear synaptopathy that selectively impairs high threshold, low-SR AN fibers and result in enhanced central gain could reproduce this pattern of improved discrimination at moderate levels but decreased performance at high levels, providing a link between peripheral pathology and central plasticity.

The mechanisms of central gain enhancement remain to be completely elucidated; however, several lines of evidence suggest that a combination of increased excitatory neuronal function and, in particular, decreased inhibitory function contribute to this experience-dependent plasticity (Yang et al., 2011; Auerbach et al., 2014; Resnik and Polley, 2017; Balaram et al., 2019). Recent studies using optogenetic manipulation (Resnik and Polley, 2017) or chronic two-photon calcium imaging of genetically labeled PV inhibitory neuron populations in the ACx (Resnik and Polley, 2021) have demonstrated that central gain changes and perceptual restoration of detection thresholds are correlated with decreased PV-mediated inhibition in the ACx. Intriguingly, these recent studies have indicated that alterations to cortical E/I balance that help restore hearing thresholds in quiet may be actively interfering with hearing in noise. Chronic two photon imaging of putative excitatory and PV inhibitory neuronal populations in the ACx found that cochlear degeneration was associated with distinct forms of plasticity in cortical excitatory and inhibitory neurons, with near complete recovery in sound-evoked responses for cortical excitatory neurons but a persistent decrease in PV neuron activity (Figure 5Ci) (Resnik and Polley, 2021). The combined effect of these changes was an increase in cortical gain that corresponded with recovery of tone detection in quiet, but also an imbalance in spontaneous activity rates between excitatory and PV inhibitory neurons that led to random surges of correlated activity that impaired tone-detection in background noise. Interestingly, combined behavioral and imaging sessions demonstrated that impairments to tone-in-noise detection following cochlear degeneration was not only the result of diminished evoked activity to target sounds but increased sensitivity to background noise, so that signal-to-noise ratios were decreased for foreground sounds (Figure 5Cii). Indeed, perceptual misses in noise were better predicted by levels of neural synchronization during the pre-stimulus period than the size of stimulus-evoked responses (Resnik and Polley, 2021). Thus, diminished PV neuron-mediated inhibition in ACx following hearing loss may be responsible for both adaptive recovery of sound detection in quiet as well as impaired adaption to background noise that disrupts perception in more challenging conditions. This degraded ability to adapt to noisy conditions could reflect impairments in bottom-up contrast gain adaptation, altered top-down modulation of cortical inhibitory circuits that act to reduce spontaneous activity during behavioral engagement, or a combination of the two.

### Hearing Loss and Top-Down Cognition

In addition to having sizeable impact on bottom-up sound processing and adaptation, hearing loss is also likely to affect top-down regulation of sound feature encoding. There is a well-characterized relationship between hearing loss and cognitive decline, although the directionality and mechanisms are strongly debated (Lin et al., 2013). It is not clear if cognitive decline or age-related hearing loss precede one another or if any such effects would even be generalizable more broadly across individuals. We will not review this debate here, except to acknowledge that cognitive decline impacts cortical circuits essential for attentional sound processing. As discussed previously, top-down circuits are critical for segregating attended streams in complex environments and reductions in cognitive capacity can alter performance in auditory scene analysis. Cognitive decline is a near universal phenomenon associated with normal aging with decline levels highly correlated with age (Park et al., 2003). Older adults are more influenced by the presence of sensory perceptual conflicts during tasks of focused attention and this coincides with reduced measures of conflict in fronto-parietal ERP markers associated with greater attentional control (Passow et al., 2014). One potential source for this reduction in performance is diminished contextual adaptation of sound level statistics within the listening environment. Herrmann et al. (2018) found that older listeners exposed to a sound distribution with two-levels showed similar neural response magnitudes but reduced capacity for sensory adaptation relative to young listeners. This finding suggests reduced capacity for adaptation to the statistical properties of the context and impaired ability to filter unattended auditory streams. While much more work is needed to elucidate the relationship between hearing loss, cognitive decline, and auditory scene analysis, current evidence suggests that hearing impairments that arise with age are likely the combined effect of disruptions to bottom-up sound processing and top-down auditory attentional regulation.

## Conclusion

We live in a world full of sounds. The auditory system employs a variety of adaptive coding strategies (Figures 1, 2) to navigate this cacophonous environment, including: compensatory dynamic range and gain adaptations to incoming stimulus statistics in order to build level and contrast invariant tuning of sound features under different background conditions (Rabinowitz et al., 2013); adaptive spatial tuning for localizing and focusing on specific sound sources to aid in the segregation of auditory streams in the presence of complex sound environment (Reed et al., 2020); and top-down attentional mechanisms that modulate auditory response and receptive field properties to selectively amplify behaviorally relevant sound features (Fritz et al., 2005b). These adaptations are observed throughout the ascending and descending auditory hierarchy to various degrees and can be both rapid, as seen in task-engaged subjects in perceptual decision-making paradigms, as well as sustained, as seen with long-term changes to auditory input associated with hearing loss.

A number of synaptic and circuit mechanisms are used to implement adaptive coding strategies in the auditory system (Figure 3), including: use-dependent changes in synaptic transmission; regulation of E/I balance to modulate response gain and minimize the influence of background noise; and top-down disinhibitory circuit motifs that can selectively modify sound encoding in response to changes in behavioral state. Interestingly, it appears that bottom-up and top-down gain changes are mediated by distinct mechanisms, suggesting the individual contributions of these different forms of adaptation are at a minimum additive or perhaps even work synergistically to enhance performance in challenging auditory scenes. Future work must investigate this possibility further by comparing neurophysiological adaptations to sound statistics in passively listening versus task-engaged animals in combination with *in vivo* manipulation of putative generators of bottom-up and top-down adaptations. It is also possible that different forms of auditory plasticity can interfere with each other, as may be the case with sensorineural hearing loss.

Listening in noisy environments poses additional challenges for those with hearing loss (Figure 4). This difficulty is due in part to degraded encoding of incoming stimuli, leading to impoverished representation of spectrotemporal sound features and disrupted ability to segregate sound sources based on select features. However, recent evidence suggests that compensatory plasticity mechanisms that help restore rapid signal detection following loss of afferent drive may actively interfere with the auditory system’s ability to adapt to more challenging listening conditions as well. For instance, increased central auditory excitability following hearing loss allows for the amplification of diminished sound-driven input from the periphery, but it may also make the auditory system more sensitive to the influence of background sounds and impair adaptation to noisy environments (Figure 5). A future challenge will be to identify whether central gain enhancement seen with hearing loss reflects bottom-up gain adaptations in response to changes in sound level statistics, reduced top-down modulation of cortical inhibitory circuits that coincide with disruptions in attentional mechanisms, or some interaction between these components.

In summary, the studies reviewed here indicate that the auditory system is highly adaptive, modulating its response properties to best fit the current environmental and/or behavioral goals. These adaptations appear to be crucial for optimal representation of sounds under diverse conditions and for listening in complex auditory environments. Further understanding of the mechanisms mediating bottom-up and top-down adaptations to sound processing, as well as the interaction between them, is crucial for harnessing the auditory system’s vast potential to compensate for difficult listening conditions, particularly following sensorineural hearing loss.

## Author Contributions

BA and HG wrote the manuscript together and approved of the submitted version.

## Conflict of Interest

The authors declare that the research was conducted in the absence of any commercial or financial relationships that could be construed as a potential conflict of interest.

## Publisher’s Note

All claims expressed in this article are solely those of the authors and do not necessarily represent those of their affiliated organizations, or those of the publisher, the editors and the reviewers. Any product that may be evaluated in this article, or claim that may be made by its manufacturer, is not guaranteed or endorsed by the publisher.
